# Constitutively-active androgen receptor variants function independently of the HSP90 chaperone but do not confer resistance to HSP90 inhibitors

**DOI:** 10.18632/oncotarget.975

**Published:** 2013-04-25

**Authors:** Joanna L. Gillis, Luke A. Selth, Margaret M. Centenera, Scott L. Townley, Shihua Sun, Stephen R. Plymate, Wayne D. Tilley, Lisa M. Butler

**Affiliations:** ^1^ Dame Roma Mitchell Cancer Research Laboratories and Adelaide Prostate Cancer Research Centre, University of Adelaide and Hanson Institute, Adelaide, South Australia, Australia.; ^2^ Department of Medicine, University of Washington School of Medicine, Seattle, Washington, USA.; ^3^ Department of Urology, University of Washington School of Medicine, Seattle, Washington, USA.; ^4^ Geriatric Research, Education and Clinical Center, Veterans Affairs Puget Sound Health Care System, Seattle, Washington, USA.

**Keywords:** Androgen receptor, variant, HSP90, HSP90 inhibitor, prostate cancer

## Abstract

The development of lethal, castration resistant prostate cancer is associated with adaptive changes to the androgen receptor (AR), including the emergence of mutant receptors and truncated, constitutively active AR variants. AR relies on the molecular chaperone HSP90 for its function in both normal and malignant prostate cells, but the requirement for HSP90 in environments with aberrant AR expression is largely unknown. Here, we investigated the efficacy of three HSP90 inhibitors, 17-AAG, HSP990 and AUY922, against clinically-relevant AR missense mutants and truncated variants. HSP90 inhibition effectively suppressed the signaling of wild-type AR and all AR missense mutants tested. By contrast, two truncated AR variants, AR-V7 and ARv567es, exhibited marked resistance to HSP90 inhibitors. Supporting this observation, nuclear localization of the truncated AR variants was not affected by HSP90 inhibition and AR variant:HSP90 complexes could not be detected in prostate cancer cells. Interestingly, HSP90 inhibition resulted in accumulation of AR-V7 and ARv567es in both cell lines and human tumor explants. Despite the apparent independence of AR variants from HSP90 and their treatment-associated induction, the growth of cell lines with endogenous or enforced expression of AR-V7 or ARv567es remained highly sensitive to AUY922. This study demonstrates that functional AR variant signaling does not confer resistance to HSP90 inhibition, yields insight into the interaction between AR and HSP90 and provides further impetus for the clinical application of HSP90 inhibitors in advanced prostate cancer.

## INTRODUCTION

The mechanisms underlying the development of castration-resistant prostate cancer (CRPC), characterized by the survival and proliferation of prostate cancer cells following androgen deprivation therapy (ADT), have been the subject of intense scrutiny over the past decade. The totality of research in this field has demonstrated that CRPC is characterized by sustained androgen receptor (AR) signaling within the “castrate” environment [1] through adaptive mechanisms such as increased intra-tumoral androgen biosynthesis [2], AR overexpression and amplification [3, 4] and increased response to or levels of AR co-regulators [5]. Another important mechanism driving castration-resistance is mutation of the *AR* gene, the frequency of which increases with tumor stage and in CRPC [6-8]. Functional analyses have demonstrated that the majority of these mutations do not cause loss of function but rather confer one of two main phenotypes: increased promiscuity of activation by non-classical ligands, or greater transactivation capacity via altered interaction with co-regulators. Archetypal examples include the T877A mutation, which is present in the LNCaP cell line and allows promiscuous activation by a variety of hormonal ligands [9], and the E235G mutation (E231G in mice), which increases basal receptor activity, affects co-regulator binding and yields a receptor that can cause oncogenic transformation of the prostate [10].

More recently, the isolation of constitutively active, truncated forms of the AR has revealed another mechanism underlying persistent AR signaling in CRPC. These so-called AR variants (ARVs), which arise due to aberrant splicing and/or structural rearrangements of the AR gene [11, 12], have variable structures but each lacks all or a portion of the ligand-binding domain (LBD) [13]. Loss of the LBD produces transcription factors that can signal in the absence of ligand and are therefore resistant to LBD-targeting AR antagonists or agents that repress androgen biosynthesis [12, 14-16]. Two of the most commonly occurring variants, ARv567es and AR-V7, are induced by castration and their expression in bone metastases of men with CRPC is associated with a particularly poor prognosis [12, 17, 18]. These observations suggest that ARVs represent an adaptive response to ADT by enabling sustained growth-promoting signaling in an androgen-deplete environment. A mechanism potentially underlying the association of ARVs with lethal disease was recently elucidated by Hu and colleagues, who showed that ARVs direct the expression of a transcriptome that is characterized by genes involved in mitosis and rapid progression through DNA-repair check points [19].

The realization that AR signaling is maintained in CRPC has underpinned the clinical development and recent FDA approval of agents that more effectively target androgen biosynthesis (e.g. abiraterone acetate) or the AR LBD (e.g. MDV3100/enzalutamide). While abiraterone and enzalutamide have improved the clinical outlook of men with CRPC, they are not curative [20, 21]. As with earlier forms of ADT, resistance to these newer generation agents may involve the emergence of novel forms of the AR, including point mutants and truncated variants [19]. As such, there is an urgent requirement for novel therapeutic strategies for CRPC that effectively inhibit all forms of aberrant AR signaling.

Heat shock protein 90 (HSP90) is an ATP-dependent molecular chaperone required for the stabilization and correct folding of > 200 proteins [22]. These “clients” include AR and a range of oncoproteins involved in diverse cellular pathways, making it an attractive target for prostate cancer [23, 24]. Moreover, HSP90 is frequently elevated in malignant prostate tissue compared to normal epithelium, highlighting its clinical relevance [25]. A number of recent studies have demonstrated the pre-clinical efficacy of HSP90 inhibitors in prostate cancer, including an ability to delay castration-resistant tumor growth [26-29]. The most extensively characterized HSP90 inhibitors are the ansamycin derivatives, including 17-allylamino-17 demethoxygeldanamycin (17-AAG) and 17-(dimethylaminotheyl-amino)-17-demethoxygeldanamycin (17-DMAG), which have performed poorly in the clinic due to poor solubility and pharmacokinetics and hepatotoxicity [30, 31]. Newer-generation agents such as NVP-AUY922 (hereafter referred to as AUY922), a resorcinylic isoxazole amide, and NVP-HSP990 (HSP990), an orally available aminopyrimidine, possess more favourable pharmacological properties and are currently being assessed in multiple clinical trials (www.clinicaltrials.gov).

Despite the potential of HSP90 inhibitors for the treatment of prostate cancer, the consequence of HSP90 inhibition has not been comprehensively assessed in the context of AR signaling by aberrant forms of the receptor, such as gain-of-function missense mutants and constitutively-active variants lacking the LBD. This is of particular relevance given the emerging realization that HSP90 and other chaperones act to stabilize mutant oncoproteins that are characteristic of many human cancers [23, 24]. In this study, we examined the efficacy of HSP90 inhibitors against a diverse range of androgen receptor mutants and variants. Our work further highlights the clinical potential of these agents in prostate cancer and provides insight into the chaperone requirements of aberrant androgen receptors.

## RESULTS

### HSP90 inhibitors repress the transcriptional activity of wild-type AR and a wide range of clinically-relevant AR mutants

**Figure 1:**
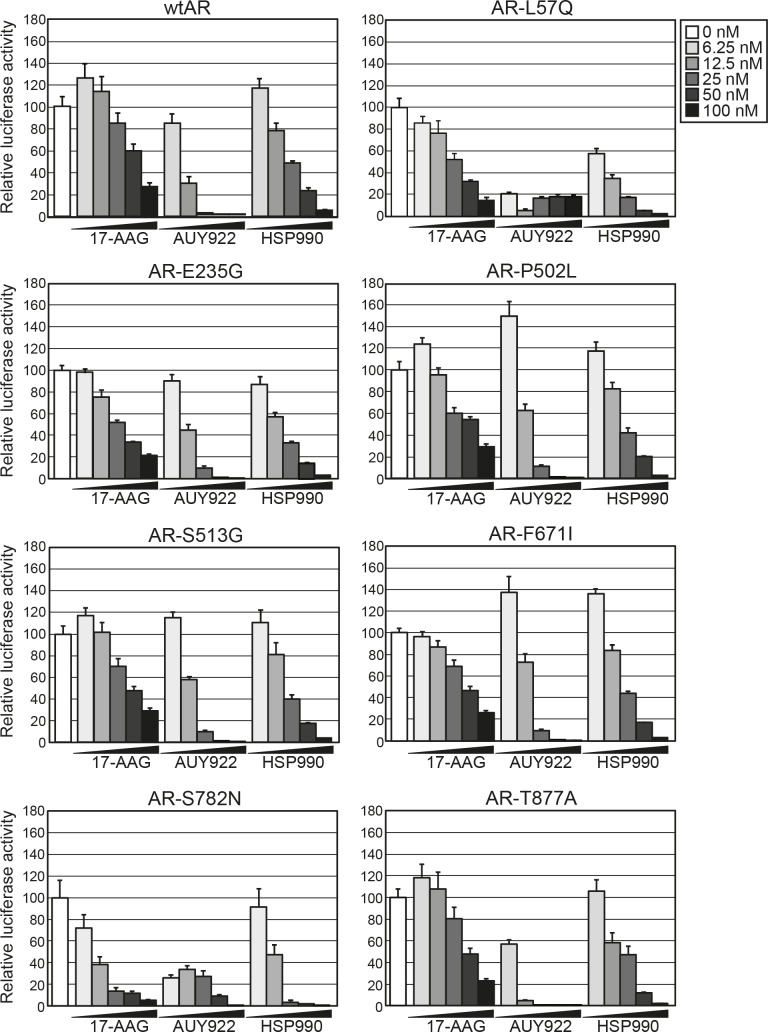
HSP90 inhibitors dose-dependently reduce transactivation activity of wtAR and gain-of-function missense mutants PC-3 cells were transfected with plasmids expressing wtAR or AR mutants and a probasin-luciferase reporter for 4 h prior to a 20 h treatment with 1 nM DHT. The doses of HSP90 inhibitor are shown to the right of the graphs. Luciferase activity values are expressed relative to 0 nM HSP90 inhibitor (set to 100%) and represent the mean (± SEM) of two independent experiments.

HSP90 inhibitors, 17-AAG, AUY922 and HSP990, were assessed for their ability to decrease wild-type AR (wtAR) transactivation activity by luciferase reporter assay in transfected PC-3 prostate cancer cells. All three HSP90 inhibitors dose-dependently decreased wtAR transactivation activity but AUY922 was the most potent agent, reducing wtAR transactivation activity by >98% at doses of 25nM and above (Fig. 1, top left). The efficacy of the HSP90 inhibitors were then assessed in cells transfected with a range of somatic missense gain-of-function AR point mutants that have been previously identified in clinical prostate tumors, mouse models, xenografts and cell lines (Supplementary Table 1). We first confirmed that each of the mutants were expressed at a similar level to wtAR and exhibited comparable DHT-dependent transcriptional activity (Supplementary Fig. 1). In response to HSP90 inhibitor treatment, all 7 AR mutants displayed a dose-dependent decrease in transactivation activity (Fig. 1). As observed for wtAR, AUY922 was consistently the most potent HSP90 inhibitor, reducing transactivation activity of all assayed AR mutants by 60-90% at ≥ 25 nM dose.

### The transcriptional activity of AR variants lacking the LBD is resistant to HSP90 inhibition

**Figure 2:**
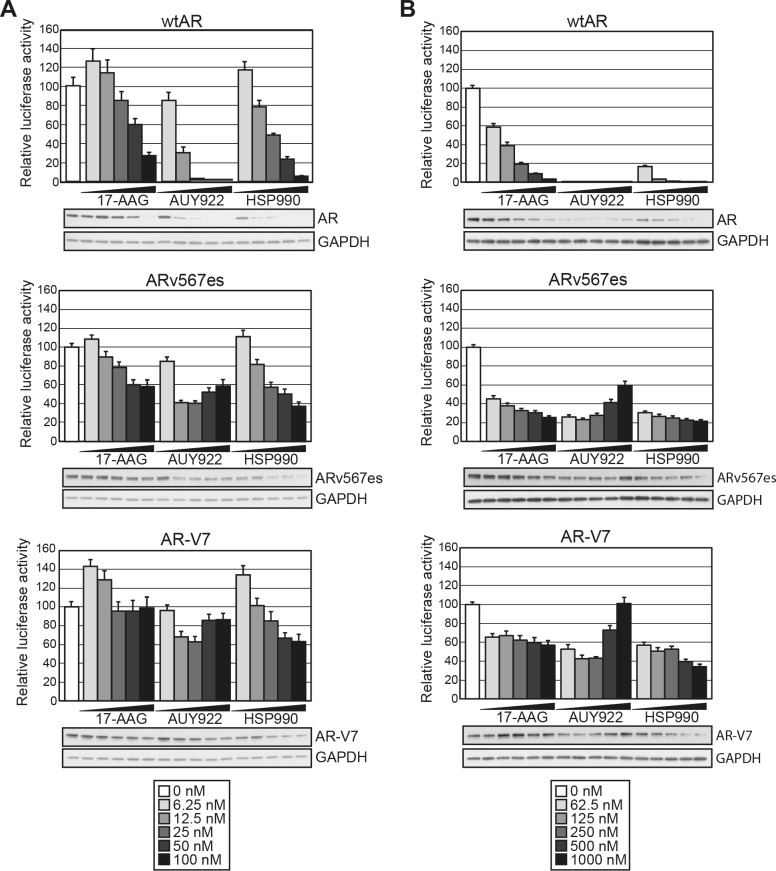
Truncated AR variants exhibit resistance to HSP90 inhibitors Transactivation assays were performed as described in Figure 1 with low doses of HSP90 inhibitors *(A)* or high doses of HSP90 inhibitors *(B)* (keys are shown below the graphs). Lysates not analyzed for luciferase activity were pooled from replicate wells and immunoblotted for AR and GAPDH to visualize steady-state protein levels.

We next assessed the effect of HSP90 inhibition on transcriptional signaling by the two most clinically relevant ARVs, ARv567es and AR-V7. Both of these variants lack the LBD, are active in the absence of androgen, and are resistant to drugs targeting the LBD [12, 19]. As expected, in our experimental system the variants could activate the synthetic probasin promoter in an androgen-independent manner (Supplementary Fig. 2). Interestingly, both truncated AR variants exhibited marked resistance to HSP90 inhibition compared to the full-length receptor (Fig. 2A). More specifically, doses up to 100 nM of 17-AAG had no effect on AR-V7 transactivation and no dose of AUY922 or HSP990 reduced either ARv567es or AR-V7 by more than ~ 60%.

Inhibition of HSP90 function is known to induce proteosomal degradation of HSP90 client proteins, including AR [32]. To determine whether the divergent responses to HSP90 inhibition were associated with changes in steady-state protein levels, lysates utilized in the luciferase assays were analyzed by immunoblotting. Protein levels of wtAR decreased in a dose-dependent manner in response to HSP90 inhibition and generally corresponded well with transcriptional activity (Fig. 2A). By contrast, steady-state protein levels of the variants were reduced by HSP90 inhibition but not to the extent of wtAR. Furthermore, in some cases robust transcriptional activity could be observed even when variant protein was effectively reduced by HSP90 inhibition (see, for example, response of AR-V7 and ARv567es to 100 nM doses of HSP990).

To assess the extent to which the constitutively-active AR variants were resistant to HSP90 inhibition, the transactivation assays were repeated at 10-fold higher doses of HSP90 inhibitor (0-1000 nM). No transcriptional activity by wtAR was observed for any AUY922 dose or any HSP990 treatment ≥ 250nM, an observation that mirrored receptor protein levels (Fig. 2B). The reduction in wtAR activity and protein was not due to a decrease in cell number within the timeframe of the experiment, as assessed by crystal violet assay (Supplementary Fig. 3). Interestingly, even with high doses of the HSP90 inhibitors, ARv567es and AR-V7 exhibited robust transcriptional activity: no dose of any HSP90 inhibitor reduced AR-V7 activity more than ~ 60%, whereas ARv567es was slightly more sensitive but still maintained > 20% of its normal activity at the highest doses (Fig. 2B). Moreover, we noted that treatment with doses of AUY922 greater than 250 nM resulted in a modest but reproducible recovery of ARV transactivation activity that was associated with increased protein levels (Fig. 2B).

### ARVs function independently of the HSP90 chaperone system

**Figure 3:**
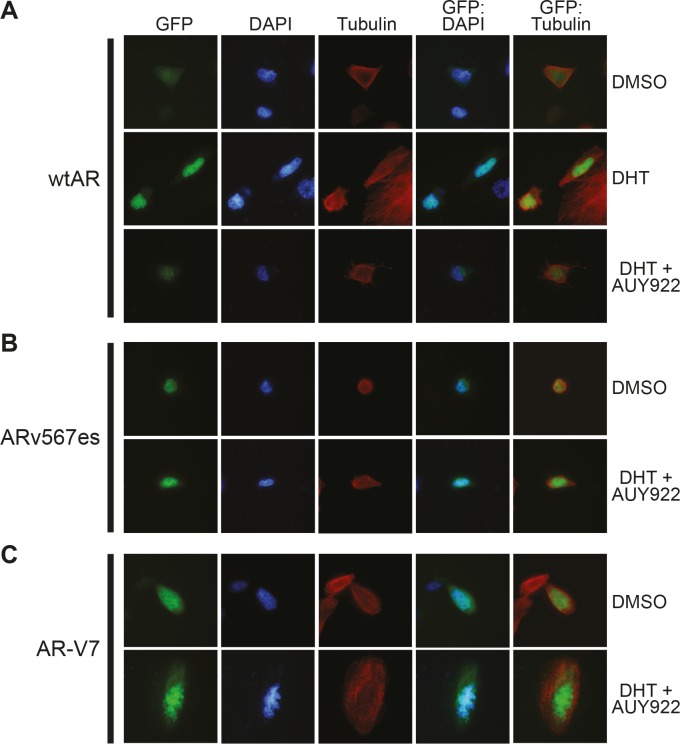
HSP90 inhibition does not affect the nuclear localization of truncated AR variants PC-3 cells grown in androgen-depleted media (phenol-red free RPMI + DCC-FBS) were transfected with GFP-tagged forms of wtAR *(A)*, AR v567es *(B)* or AR-V7 *(C)* and treated with DMSO (vehicle control), 1 nM DHT or 1 nM DHT and 100 nM AUY922. Nuclei were stained with DAPI and cytoskeletons with tubulin. Representative images are shown for five color channels (GFP, DAPI, tubulin, merged GFP/DAPI and merged GFP/tubulin).

To further investigate the apparent resistance of ARv567es and AR-V7 to these agents, we determined whether HSP90 inhibition affected the subcellular localization of GFP-tagged forms of the variants. We first confirmed that GFP-wtAR, GFP-AR-V7 and GFP-ARv567es were transcriptionally active in PC-3 cells (Supplementary Fig. 4A). Subsequently, cells expressing the fusion proteins were treated with DHT and/or AUY922 and fluorescence monitored after 48 h. Wild-type AR was primarily cytoplasmic when cells were grown in the absence of androgen (charcoal-stripped serum) and shifted rapidly to the nucleus with addition of DHT (Fig. 3A). However, co-treatment with AUY922 blocked the DHT-mediated nuclear import of wtAR (Fig. 3A). By contrast, AR-V7 and ARv567es exhibited primarily nuclear localization in the absence of DHT and this distribution was not altered when cells were treated with AUY922 (Figs. 3B and 3C). Thus, nuclear import of ARVs appears to be independent of the HSP90 chaperone system.

**Figure 4:**
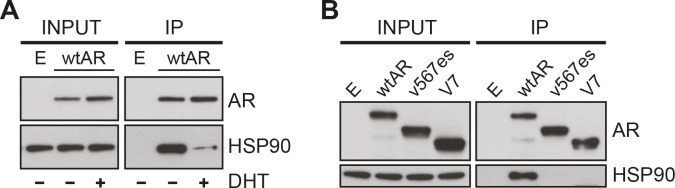
Truncated AR variants do not form stable complexes with HSP90 *(A)* DHT promotes dissociation of wild-type AR from HSP90. PC-3 cells grown in androgen-depleted media (phenol-red free RPMI + DCC-FBS) were transfected with FLAG-tagged wtAR or empty plasmid and treated with 1 nM DHT or vehicle control. FLAG-tagged wtAR protein complexes were immunoprecipitated and immunoblotted for HSP90. *(B)* Truncated AR variants do not associate with HSP90. PC-3 cells grown in androgen-depleted media were transfected with FLAG-tagged wtAR, ARv567es or AR-V7 or an empty plasmid control and grown in an androgen-depleted environment. FLAG-tagged AR protein complexes were immunoprecipitated and immunoblotted for HSP90.

To further examine the relationship between ARVs and HSP90, we tested whether ARV:HSP90 protein complexes exist in prostate cancer cells by co-immunoprecipitation assays. FLAG-tagged forms of wtAR and the ARVs, which exhibited normal transcriptional activity (Supplementary Fig. 4B), were used in this experiment. FLAG-AR was isolated by immunoprecipitation under non-denaturing conditions, and molybdate was included to preserve chaperone interactions [33]. As expected, wtAR formed a complex with HSP90 when grown in androgen-depleted media that was largely lost following the addition of 1 nM DHT (Fig. 4A). By contrast, neither of the AR variants co-immunoprecipitated detectable amounts of HSP90 in androgen-depleted media (Fig. 4B), further suggesting that classical AR regulatory mechanisms do not influence ARV function.

### ARV expression is induced by HSP90 inhibition

**Figure 5:**
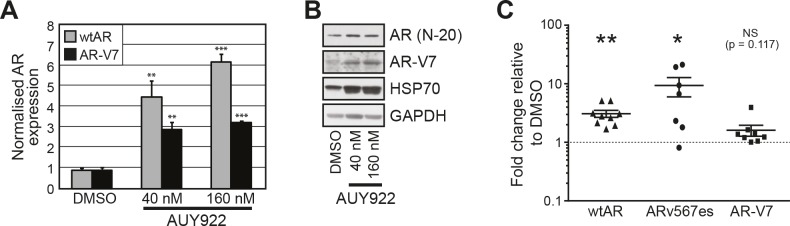
AUY922 modulates the levels of wtAR and AR-V7 transcript and protein *(A)* VCaP cells were treated for 24 h with the indicated doses of AUY922 or vehicle DMSO. Wild-type AR and AR-V7 mRNA was measured by quantitative RT-PCR (normalized to reference gene GAPDH; DMSO set to 1). Values are the mean (± SEM) of triplicate samples; results are representative of two independent experiments. *(B)* Lysates from cells treated in part A were collected and immunoblotted for wtAR (AR N-20; AR C-19), HSP70 or AR-V7. GAPDH was used as a loading control. *(C)* Human prostate tumor explants were cultured in medium containing 500 nM AUY922 or vehicle DMSO for 48 h. Wild-type AR, AR-V7 and ARv567es mRNA was measured by quantitative RT-PCR (normalized to reference gene GAPDH; DMSO set to 1). Statistically significant differences compared to DMSO control treatment were assessed using one-sample *t* tests (*, *p* < 0.05; **, *p* < 0.01).

Our results suggest that transiently-expressed ARVs function largely independently of the HSP90 chaperone. To assess whether HSP90 inhibitors affect the expression and/or stability of endogenously-expressed ARVs, protein and RNA was isolated from VCaP cells that had been treated for 24 h with AUY922. VCaP cells were chosen because they express both AR-V7 and ARv567es and exhibit selection of AR-V7 in response to agents targeting the LBD (e.g. MDV3100) [19]. qRT-PCR quantification revealed that full-length AR (detected with primers that amplify a region crossing the exon 5-6 splice junction) and AR-V7 expression increased in response to 40 and 160 nM AUY922 treatment compared to vehicle control (DMSO) (Fig. 5A). In our hands, ARv567es mRNA was not detectable by Taqman probe-based qRT-PCR in this cell line (data not shown), possibly because cells were grown in media containing full serum and supplemented with 0.1% DHT, which is known to repress ARv567es expression [19]. Corresponding protein lysates were immunoblotted for AR to assess whether the observed changes in mRNA were coupled to an increase in protein level (Fig. 5B). Induction of HSP70, a known marker of HSP90 inhibition [26], confirmed the efficacy of the drug treatments (Fig. 5B). Importantly, AR-V7 protein levels were robustly increased following treatment with AUY922. Interestingly, loss of wtAR protein was not observed by HSP90 inhibition in VCaP cells, a response that is incongruent with other prostate cancer cell lines (e.g. LNCaP, C4-2B, 22Rv1) and tumor explants [26, 27, 34, 35].

To investigate the relationship between HSP90 inhibition and ARV expression in a more biologically relevant system, we evaluated AR and ARV expression in human prostate tumors cultured *ex vivo* in medium containing AUY922. As previously observed [26], treatment of tumor tissue with AUY922 markedly reduced proliferation, as measured by Ki67 immunohistochemistry (Supplementary Fig. 5). We have previously demonstrated that AUY922 decreases wtAR protein levels in primary tumors cultured in this manner [26]. By contrast, wtAR mRNA levels increased in 9/9 tumors (average = 3.1-fold) in response to treatment with 500 nM AUY922 compared to vehicle control (DMSO) (Fig. 5C) (one sided *t* test, p = 0.0011). ARV expression was also induced by AUY922: ARv567es (detectable in 7/9 tumours) and AR-V7 (detectable in 8/9 tumors) transcript levels increased by an average of 8.8-fold (p = 0.0493) and 1.6-fold (p = 0.1170), respectively (Fig. 5C). These data reveal that in *ex vivo* cultured primary tumors, AUY922 induces full-length AR and ARV mRNA expression concomitantly with inhibition of proliferation.

### Growth of ARV-expressing prostate cancer cell lines is suppressed by HSP90 inhibition

**Figure 6:**
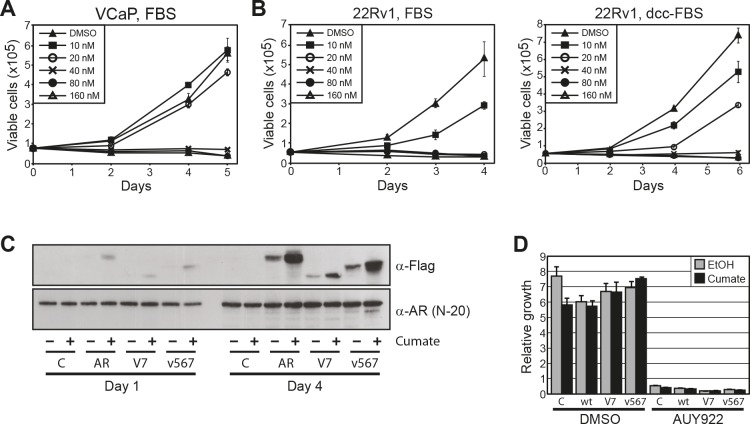
Prostate cancer cells expressing ARVs are sensitive to HSP90 inhibition *(A)* AUY922 reduces cell viability of VCaP prostate cancer cells. Proliferation of cells cultured with increasing concentrations of AUY922 was assessed by trypan blue exclusion assays. Cells were grown in media containing 10% FBS + 0.1 nM DHT. *(B)* AUY922 reduces cell viability of 22Rv1 prostate cancer cells, as assessed by trypan blue exclusion assays. Cells were grown in the presence of androgen (10% FBS; left) or in androgen-depleted media (10% DCC-FBS; right). *(C)* Induction of FLAG-ARVs by cumate in stably-transduced LNCaP cells. Western blots with FLAG antibody (M2; top) or AR antibody (N-20; bottom) are shown. *(D)* AUY922 reduces cell viability of LNCaP cells stably transfected with inducible expression constructs: empty (C), wtAR (wt), AR-V7 (V7) and ARv567es (v567). Cells were cultured in 10% FBS and treated with cumate to induce protein expression or ethanol control (EtOH), followed by AUY922 or DMSO (vehicle control). Counts were performed as described above.

Given that ARVs are resistant to HSP90 inhibitors at a transcriptional level, can enter the nucleus independently of the HSP90 chaperone system and can be induced by AUY922 in primary tumors, we reasoned that persistent ARV-mediated signaling in prostate cancer cells could confer some level of resistance to HSP90 inhibition. To test this idea, we assessed the growth-suppressive activity of AUY922 in the VCaP line. As reported previously [26], these cells were sensitive to AUY922 treatment, with maximal anti-proliferative activity observed at 40 nM (Fig. 6A). VCaP cells did not grow well in androgen-deplete media and growth suppression by AUY922 could not be assessed under these conditions (data not shown).

We subsequently investigated the efficacy of AUY922 in another prostate cancer cell line with endogenous ARV expression, 22Rv1 [14]. The ability of 22Rv1 cells to grow in the absence of androgen or in the presence of enzalutamide (MDV3100) is conferred by expression of the AR-V7 variant [14, 36]. Cell proliferation was reduced in a dose-dependent manner by AUY922 in both androgen-replete (full fetal bovine serum (FBS)) and androgen-deplete (charcoal-stripped serum; DCC-FBS) media (Fig. 6B), where maximal inhibition was observed at 20 nM and 40 nM, respectively.

To more directly investigate the potential of ARVs to promote resistance to AUY922, we generated LNCaP cells stably transfected with inducible forms of wtAR, AR-V7 and ARv567es. Upon treatment with cumate, the proteins were expressed at levels approximately 1-5% of the endogenous wtAR (Fig. 6C), a ratio that has been reported to be physiologically relevant [17]. In growth assays, robust induction of the proteins was observed after 4 days (Fig. 6C). As expected, AUY922 effectively suppressed the growth of LNCaP cells expressing physiological levels of wtAR (control line + ethanol/cumate; wtAR line + ethanol), and over-expression of wtAR following cumate treatment had no discernable impact on drug efficacy (Fig. 6D). Similarly, enforced over-expression of AR-V7 or ARv567es did not confer any detectable level of resistance to HSP90 inhibition by AUY922 (Fig. 6D).

## DISCUSSION

Current treatments for CRPC prolong survival, but are not curative. The critical role of HSP90 in the stabilization, maturation and activation of AR and other proteins that contribute to the progression of prostate cancer highlights the potential of HSP90 inhibitors as therapeutic agents. However, until now, no study has comprehensively assessed whether clinically relevant gain-of-function AR mutants and truncated androgen receptors that can emerge in CRPC are reliant on the HSP90 chaperone system for activity.

Using AR-specific reporter assays, we found that HSP90 inhibition effectively disrupted the transcriptional activity of a wide range of clinically-relevant AR somatic missense point mutants. Therefore, as for wtAR, canonical HSP90 regulatory pathways are likely to control the maturation, stabilization, ligand binding and nuclear import of mutant receptors. This finding strengthens the case for the application of HSP90 inhibitors in castration-resistant tumors, which are more likely to express gain-of-function mutant receptors. As expected from IC_50_ values, solubility and affinities for HSP90, AUY922 and HSP990 were more efficacious against the AR mutants than the ansamycin derivative, 17-AAG. This corresponds with previous work from our laboratory demonstrating enhanced efficacy of AUY922 and HSP990 in the LNCaP cell line that expresses the T877A mutation [26]. The increased capacity of these new generation agents to disrupt both wild-type and mutant AR signaling, in addition to their improved pharmacologic and toxicologic profiles, further supports their clinical investigation in men with advanced prostate cancer.

In contrast to the missense AR mutants, the C-terminally truncated AR-V7 and ARv567es both displayed resistance to HSP90 inhibitors at a transcriptional level. Indeed, even doses up to 1000 nM of AUY922 and HSP990 only partially disrupted ARV signaling. Similarly, a recent study found that the transcriptional activity of AR-V7 was shown to be resistant to other new and old-generation HSP90 inhibitors (ganetespib and geldanamycin, respectively) [37]. Two findings from our study likely underpin the apparent independence of ARV activity from HSP90. First, the nuclear localization of AR-V7 and ARv567es was largely unaffected by the potent HSP90 inhibitor, AUY922. This observation mirrors recent research demonstrating that the nuclear import of ARVs was not disrupted by 17-AAG [38]. By contrast, even in a cell line model of CRPC that exhibits ligand-independent activity of full-length AR, normal HSP90 function is necessary for nuclear import of the receptor [39], highlighting the distinction between ARVs and wtAR. Second, co-immunoprecipitation assays revealed that interactions between ARVs and HSP90 are not detectable even in cells transiently expressing high levels of the ARV. Interaction surface mapping has demonstrated that chaperone interactions localize primarily to the LBD of steroid receptors [22]. More specifically, analysis of the rat AR found that amino acids 703 and 758 of the rat AR, corresponding to the hinge region and part of the LBD in the human AR, are important for its interaction with HSP90 [40]. Studies of the glucocorticoid receptor identified a specific 7-amino acid sequence required for HSP90 binding that corresponds with amino acids 670 to 676 in the hinge domain of the human AR [41], which is a reported protein-protein interaction surface [42]. ARv567es contains the hinge domain but none of the LBD whereas AR-V7 lacks both the hinge and LBD, supporting a role for these C-terminal domains in mediating an interaction between full length AR and HSP90.

Collectively, the observation that HSP90 inhibitors have a minimal effect on ARV transcriptional activity and nuclear localization and our inability to detect HSP90:ARV protein complexes indicate that AR-V7 and ARv567es function largely independently of HSP90. Whether other proteins “chaperone” ARVs is currently unknown. Classic members of the HSP90 molecular machinery, such as HSP70, p23, FKBP52 and Cyp40, either bind to the AR LBD or indirectly with AR via HSP90 [22, 43]. As such, we do not expect these factors to be important regulators of C-terminally truncated AR variants. A plausible candidate ARV chaperone is HSP27, which was found to bind directly to the AR NTD *in vitro* and has been linked to treatment resistance and cell survival in prostate cancer [44]. At least two lines of evidence support the concept that a non-canonical chaperone system, perhaps comprising HSP27 and/or other factors, regulates ARV function. First, by comparing the expression of full-length AR with ARVs at both the protein and mRNA level, Hornberg and colleagues found evidence for increased stability of ARVs in CRPC bone metastases [17]. Protection from intracellular protein surveillance mechanisms, such as the ubiquitin-proteasome system, is a major function of HSPs and stress proteins. Second, the intrinsically disordered nature of the NTD means that chaperone-mediated protection from misfolding, aggregation and illicit protein:protein interactions would likely benefit ARVs. Identifying ARV regulators, including chaperones, is an important next step in elucidating the function of these novel drivers of prostate cancer progression and is likely to have significant clinical implications.

While our discussion has focused on the resistance of ARVs to HSP90 inhibition, the observation that high doses of all three agents at least partially suppressed ARV-driven transcription is worth consideration. We envision at least three possible explanations for this finding. First, we cannot rule out the possibility that ARVs retain a residual level of interaction with, and reliance on, HSP90. Indeed, a subset of the variants retain the hinge region, a proposed HSP90 interaction surface. Second, as discussed above, ARVs may utilize alternative chaperones that could be affected by high concentrations of HSP90 inhibitors. Finally, ARV activity is likely to be indirectly affected by HSP90 inhibition through deregulation of alternative client proteins. For example, it was recently found that suppression of the PI3K-AKT pathway inhibits AR-V7 transcriptional activity [45], and HSP90 is an important regulator of AKT [46].

Despite the apparent autonomy of ARVs from the HSP90 chaperone system, we observed that the growth/proliferation of cell lines expressing AR-V7 or ARv567es, either endogenously or in an enforced manner from an inducible promoter, is highly sensitive to HSP90 inhibition. Thus, the growth advantage associated with ARV expression is unable to compensate for disruption of other critical cellular pathways by HSP90 inhibitors. It follows that sensitivity to these agents is largely independent from both classical and aberrant AR signaling axes, a concept further supported by the observation that HSP90 inhibitors are highly effective in both AR-positive and AR-negative settings *in vitro* [26]. Collectively, these findings highlight the broad mode of action of these agents, a characteristic also likely to minimize the therapy-mediated selection pressures that drive drug resistance.

This study also provided insight into the relationship between HSP90 inhibition and *AR* gene expression. Importantly, we demonstrated that treatment with the potent HSP90 inhibitor AUY922 caused an increased level of transcripts encoding full-length AR (VCaP cells and primary tumors), AR-V7 (VCaP cells only; trend to increase in primary tumors) and ARv567es (primary tumors only; not detectable in VCaP cells). One potential explanation for this finding is that liganded AR can negatively regulate its own expression [47]: thus, the loss of AR protein associated with HSP90 inhibition, which occurs in AUY922-treated, *ex vivo* cultured prostate tumors [26], could de-repress the *AR* gene. This hypothesis may also explain why AR inhibition (MDV3100 treatment or knockdown with siRNA) leads to accumulation of AR-V7 and emergence of an alternative transcriptome [19]. Alternatively, HSP90 inhibition may impact on mRNA splicing, stability and/or translation, leading to accumulation of AR mRNA [48]. Irrespective of mechanism, the finding that HSP90 inhibitors can cause accumulation of AR and ARVs may be relevant to the clinical application of these agents. For example, although we have demonstrated that the broad mode of action of HSP90 inhibitors can overcome the selection for ARVs in short-term *in vitro* experiments, the consequences of such selection during prolonged treatment of men is unknown. Our findings provide a rationale for clinical application of a combinatorial treatment strategy comprising HSP90 inhibitors and agents that target the AR-NTD.

AR-V7 and ARv567es were detectable by qRT-PCR in 8/9 and 7/9 primary tumors, respectively. For AR-V7, this proportion is similar to what has been reported in previous studies [16, 17]. By contrast, ARv567es has rarely been detected in primary tumors to date [12, 17]. A possible explanation for this discrepancy relates to methodology: we employed a highly specific probe-based assay rather than the SYBR assay employed in earlier studies. Our data suggests that ARv567es may be more prevalent than previously estimated, an important finding given its association with survival [17].

In summary, our study provides key new insights into the clinical potential of HSP90 inhibitors in cells that are characterized by aberrant AR signaling. Despite the independence of constitutively-active AR variants from the HSP90 chaperone system, HSP90 inhibitors are likely to be effective in CRPC by targeting signaling by wild-type AR, missense AR mutants and other critical pathways required for growth and survival of prostate cancer cells.

## MATERIALS AND METHODS

### Reagents and antibodies

AUY922 and HSP990 were obtained from Novartis and dissolved in dimethyl sulfoxide (DMSO). 17-AAG and MDV3100 were obtained from the National Cancer Institute and Sellicks Chemicals, respectively, and dissolved in DMSO. Primary antibodies used for Western blotting were AR-N20 (1:1000; SC-816, Santa Cruz Biotechnology Inc), AR-V7 (1:500; AG10008, Precision Antibody), HSP70 (1:1000; SPA-812, Stressgen Bioreagents), HSP90α/β (1:1000; SC-7947, Santa Cruz), GAPDH (1:2000; MAB374, Millipore) and FLAG (1:2000; M2, Sigma). A tubulin antibody (1:2500; 05-829, Millipore) antibody was used for immunofluorescence.

### Plasmids

The pGL_4.14_-probasin-ARR3-tk-luciferase reporter has been reported [32]. Plasmid constructs harbouring full-length AR with somatic missense mutations were created from pCMV-AR by PCR-based megaprimer *in vitro* mutagenesis as previously described [33]. pcDNA-AR-V7 and pcDNA-ARv567es expression plasmids have been described [19]. pcDNA-HA-GFP-ARv567es was made by inserting a PCR product containing the complete GFP coding sequence into the BamHI site of pcDNA-HA-ARv567es [12]. pEGFP-AR-V7 and pEGFP-AR were generously provided by M. Marcelli [34]. pCMV-3xFLAG-AR, pCMV-3xFLAG-V7 and pCMV-3xFLAG-v567es were made by ligating AR and ARV PCR products into the EcoRI/XbaI sites of p3xFLAG-CMV-9 (Sigma). 3xFLAG-AR fragments were also inserted into the pCDH-EF1-CymR-T2A-Puro vector (System Biosciences).

### Cell lines

VCaP, PC-3 and 22Rv1 human prostate carcinoma cells were obtained from the American Type Culture Collection. All cell lines underwent verification by short-tandem repeat profiling in 2010 by CellBank Australia. PC-3 cells were maintained in RPMI-1640 containing 5% FBS or phenol-red free RPMI containing 5% charcoal-stripped serum (DCC-FBS). 22Rv1 cells were maintained in RPMI-1640 containing 10% FBS or phenol-red free RPMI containing 10% charcoal-stripped serum (DCC-FBS). VCaP cells were maintained in Dulbecco's Modified Eagle's Medium containing 10% FBS, 1% sodium pyruvate, 1% MEM non–essential amino acids, and 0.1 nM 5α-dihydrotestosterone (DHT; Sigma). LNCaP lines expressing cumate-inducible 3xFLAG-wtAR, 3xFLAG-ARv567es and 3xFLAG-AR-V7 lentivirus were made using the SparQ cumate switch lentivector system (Systems Biosciences). pCDH-EF1-CymR-T2A-Puro vectors were packaged into lentiviral particles using pPACK packaging systems (System Biosciences). To make stable cell lines, LNCaP cells were infected with 1 × 10^7^ virus particles per 1 × 10^6^ cells then selected with 1 ug/ml puromycin (Invitrogen) for 10 days. Stably transduced LNCaP lines were maintained in RPMI-1640 containing 10% FBS.

### Luciferase transactivation assays

AR transactivation assays were performed essentially as described [32]. Briefly, AR-negative PC-3 prostate cancer cells were seeded in 96-well plates (1.5 × 10^4^ cells/well) and then transfected with 100 ng of pGL_4.14_-probasin-ARR3-tk-luciferase reporter plasmid and 1.5 ng of the appropriate AR expression construct using Lipofectamine 2000 (Invitrogen). Plasmid quantities were chosen to ensure receptor activity was within the linear range. After 4 h, treatment media containing HSP90 inhibitor (0-1000 nM) and 1 nM DHT (Sigma) was added to the cells. Cells were lysed 24 h later with Passive Lysis Buffer (Promega) and frozen overnight at -80°C. Lysates were assayed for luciferase activity using the Luciferase Assay System (Promega) and a plate reading luminometer (TopCount; Packard). Subsequently, protein from six replicate wells was pooled, centrifuged at 10,000*g* for 10 min at 4°C, and equal volumes of the supernatants were analyzed by SDS-PAGE and Western blotting.

### Visualization of GFP-tagged proteins

PC-3 cells were seeded at a density of 3.0 × 10^4^ cells/well in 8-well Lab-Tek chamber slides (Nunc) and left overnight to adhere. The cells were then transfected with plasmids expressing GFP-tagged AR, AR-V7 and ARv567es using Lipofectamine 2000. After 24 h the cells were treated with AUY922 or DMSO (vehicle control) for 1 h in the absence or presence of 1nM DHT or ethanol (vehicle control). The cells were then fixed using 4% paraformaldehyde, washed with PBS and incubated overnight at 4°C with an antibody specific for tubulin. Alexa-Fluor 594 donkey anti-mouse IgG (Life Technologies) was used to visualize tubulin staining. ProLong Gold Antifade Reagent with DAPI (Life Technologies) was used as mounting media and to detect cell nuclei. Cells were viewed using an Olympus IX71 fluorescent microscope and images were obtained with an Olympus DP70 cooled digital color camera at 20x magnification.

### Co-immunoprecipitation

AR:HSP90 complexes were isolated by immunoprecipitation as described previously [35]. Briefly, androgen-starved PC-3 cells were transfected with constructs expressing FLAG-tagged wild-type AR or ARVs using Lipofectamine 2000 (Invitrogen) for 4 h. The cells were treated 24 h later with vehicle (0.1% ethanol) or 1 nM DHT (Sigma) for 1 h in 5% DCC-FBS phenol-red free RPMI, then lysed in 500 μl IP buffer (20 mM Tris HCl (pH 7.5), 50 mM NaCl, 20 mM Na_2_MoO_4_, 0.5% NP-40 Alternative, 1 mM EDTA, 1 mM EGTA (pH 8.0), 2 mM DTT and protease inhibitors). The lysates were sonicated twice at low power for 15 sec each (Bioruptor; Diagenode) and cleared by centrifugation (10 min, 16,000*g* at 4°C). Samples were immunoprecipitated for 2 h with rotation at 4°C using anti-FLAG M2 agarose beads (Sigma) and then washed three times with IP buffer. Protein was eluted by boiling in SDS-PAGE loading buffer for 5 min and analyzed by SDS-PAGE and Western blotting.

### Cell growth assays

Trypan blue exclusion counts were performed on 22Rv1 and VCaP cells treated with AUY922. Growth assays were stopped when DMSO-treated cells reached the stationary phase of growth. For LNCaP lines with inducible AR and ARV expression, cells were seeded out at 2 × 10^4^ cells/well in 24-well plates. The next day, day zero counts were taken and cells were treated with 0.3 ug/ml cumate to induce AR and ARV expression. One day later, cells were treated as appropriate with AUY922 or DMSO (control). Cells were counted after a further 72 hours using trypan blue exclusion assays.

### *Ex vivo* culture of human prostate tumors

Fresh prostate cancer specimens were obtained with written informed consent through the Australian Prostate Cancer BioResource from men undergoing robotic radical prostatectomy at the Royal Adelaide Hospital. Tissues were dissected and cultured as described previously [26] in medium containing DMSO or 500 nM AUY922. After 48 h of culture, tissues were snap frozen and stored at −80°C.

### RNA extraction and quantitative real-time RT-PCR

For cell line experiments, VCaP cells were seeded at a density of 4 × 10^5^ cells/well in 12 well plates and treated for 24 h with AUY922 (40 or 160 nM) or DMSO. RNA was extracted using Trizol using standard techniques. For prostate tumors, samples were homogenized using a Precellys tissue homogenizer (Bertin Technologies) or mortar and pestle and RNA was extracted using Trizol. 200 ng of RNA was reverse transcribed and analyzed by SYBR green qRT-PCR as described previously [36]. Primers for full-length AR (forward: CCTGGCTTCCGCAACTTACAC; reverse: GGACTTGTGCATGCGGTACTCA) amplify a region spanning exons 5 and 6, which is not found in C-terminally truncated AR variants. Primers for AR-V7 have been described previously [16]. Taqman qRT-PCR was used to quantify ARv567es using the following primers and probe (forward: CCTTGCTCTCTAGCCTCAATGAA; reverse: CTTGATTAGCAGGTCAAAAGTGAACT; probe: 6FAM-CCTTGCCTGATTGCGAGA-MGB-NFQ).

### Statistical analysis

Changes in the expression level of AR and AR-V7 in VCaP cells treated with AUY922 were assessed using one-way ANOVA with Tukey's post hoc test. Changes in the expression level of AR and ARVs in tumor explants treated with AUY922 were assessed using a one-sample *t* test (where the control treatment, DMSO, was set to the hypothetical mean of 1). All statistical analyses were done using GraphPad Prism Software.

## Supplementary Figures and Tables


