# Supplementary material for: Constitutively-active androgen receptor variants function independently of the HSP90 chaperone but do not confer resistance to HSP90 inhibitors
Source: Oncotarget. 2013 Apr 25;4(5):691–704. doi: 10.18632/oncotarget.975 (PMC3742830; doi:10.18632/oncotarget.975)
Supplement: Supplementary file 1 [file oncotarget-04-691-s001.pdf]

Supplementary Table 1. Description of AR mutations

Mutation	Origin	Effect on AR signaling	Reference
L57Q	Advanced primary tumor	Unknown	(1)
E235G	AI[1] tumor from TRAMP[2] mouse model	Increased basal activity and response to coregulators	(2)
P502L	Tumor from patient receiving complete androgen blockade	Increased response to p160 coregulators (unpublished data from Tilley laboratory)	(3)
S513G	Tumor from patient receiving complete androgen blockade	Increased response to p160 coregulators (unpublished data from Tilley laboratory)	(3)
F671I	Intact tumor from TRAMP[%] mouse model	Increased responsiveness to DHT	(2, 4)
S782N	Advanced primary tumor	Increased responsiveness to DHEA[3]	Unpublished
T877A	LNCaP cell line	Altered binding specificity	(5)

[1] Androgen-independent
[2] Transgenic adenocarcinoma of mouse prostate
[3] Dehydroepiandrosterone

References

1. Tilley WD, Buchanan G, Hickey TE, Bentel JM. Mutations in the androgen receptor gene are associated with progression of human prostate cancer to androgen independence. Clin Cancer Res. 1996;2:277-85.
2. Han G, Foster BA, Mistry S, Buchanan G, Harris JM, Tilley WD, et al. Hormone status selects for spontaneous somatic androgen receptor variants that demonstrate specific ligand and cofactor dependent activities in autochthonous prostate cancer. J Biol Chem. 2001;276:11204-13.
3. Hay CW, McEwan IJ. The impact of point mutations in the human androgen receptor: classification of mutations on the basis of transcriptional activity. PLoS One. 2012;7:e32514.
4. Buchanan G, Yang M, Harris JM, Nahm HS, Han G, Moore N, et al. Mutations at the boundary of the hinge and ligand binding domain of the androgen receptor confer increased transactivation function. Mol Endocrinol. 2001;15:46-56.
5. Veldscholte J, Berrevoets CA, Brinkmann AO, Grootegoed JA, Mulder E. Anti-androgens and the mutated androgen receptor of LNCaP cells: differential effects on binding affinity, heat-shock protein interaction, and transcription activation. Biochemistry. 1992;31:2393-9.

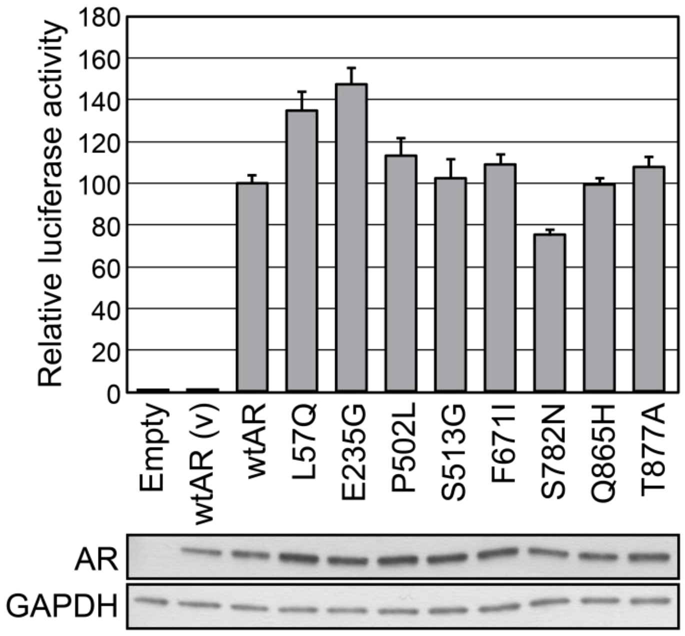

Supplementary Figure 1. Comparison of the transcriptional activity and protein expression of wtAR and AR mutants. PC-3 cells were transfected with an empty plasmid or plasmids expressing the indicated AR mutants and a probasin-luciferase reporter for 4 h prior to a 20 h treatment with 1 nM DHT (or vehicle control; v). Luciferase activity values are expressed relative to wtAR + DHT (set to 100%) and represent the mean (± SEM) of two independent experiments. Lysates not assayed for luciferase activity were pooled from replicate wells and immunoblotted for AR and GAPDH to visualize steady-state protein levels.

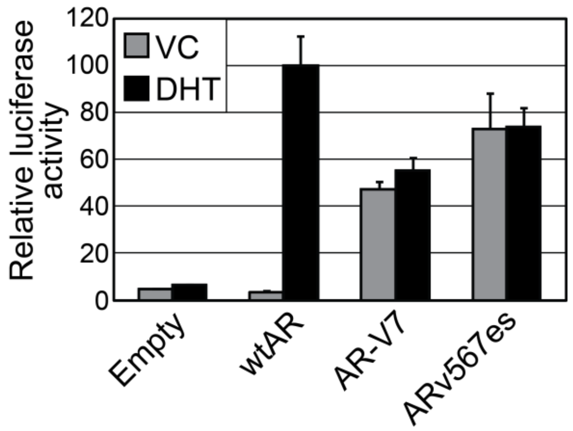

Supplementary Figure 2. Comparison of the transcriptional activity and protein expression of wtAR and truncated AR variants. Experimental set-up as described in legend of Supplementary Figure 1. VC, vehicle control (ethanol); DHT, dihydrotestosterone. Luciferase activity values are expressed relative to wtAR + DHT (set to 100%).

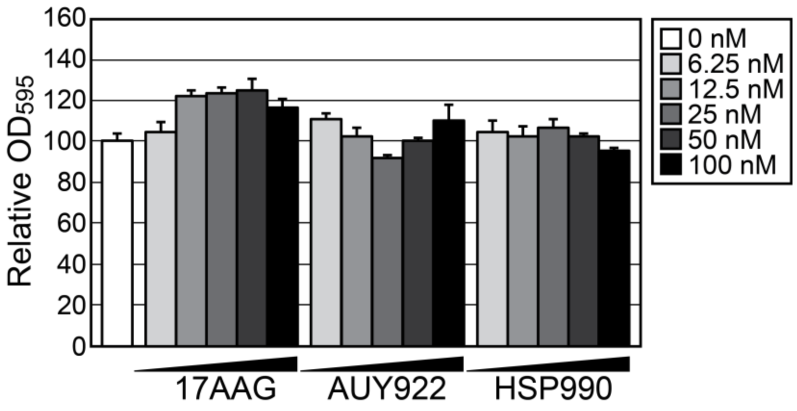

Supplementary Figure 3. Reduction of AR transactivation activity is not due to decreased cell viability. Cells treated for 20 h with 0-1000 nM HSP90 inhibitor were assessed for relative viability by crystal violet staining. Optical density (OD) at 595 nm is expressed relative to wtAR + 0 nM HSP90 inhibitor (set to 100%) and represents the mean (± SEM) of 6 replicate treatment wells in a 96-well plate.

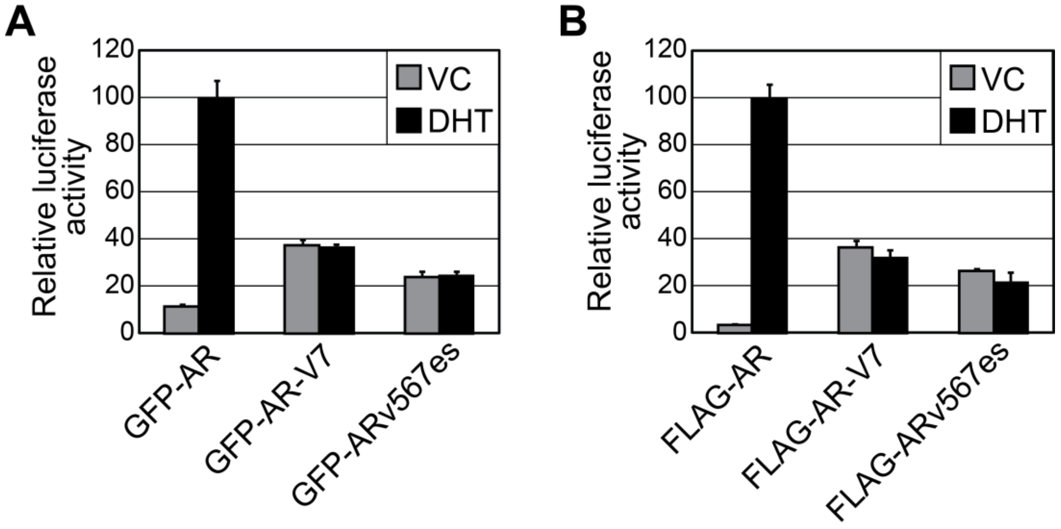

Supplementary Figure 4. GFP-tagged (A) and FLAG-tagged (B) AR proteins are transcriptionally active. Experimental set-up as described in legend of Supplementary Figure 1. Luciferase activity values are expressed relative to AR + DHT (set to 100%). VC, vehicle control (ethanol); DHT, dihydrotestosterone.

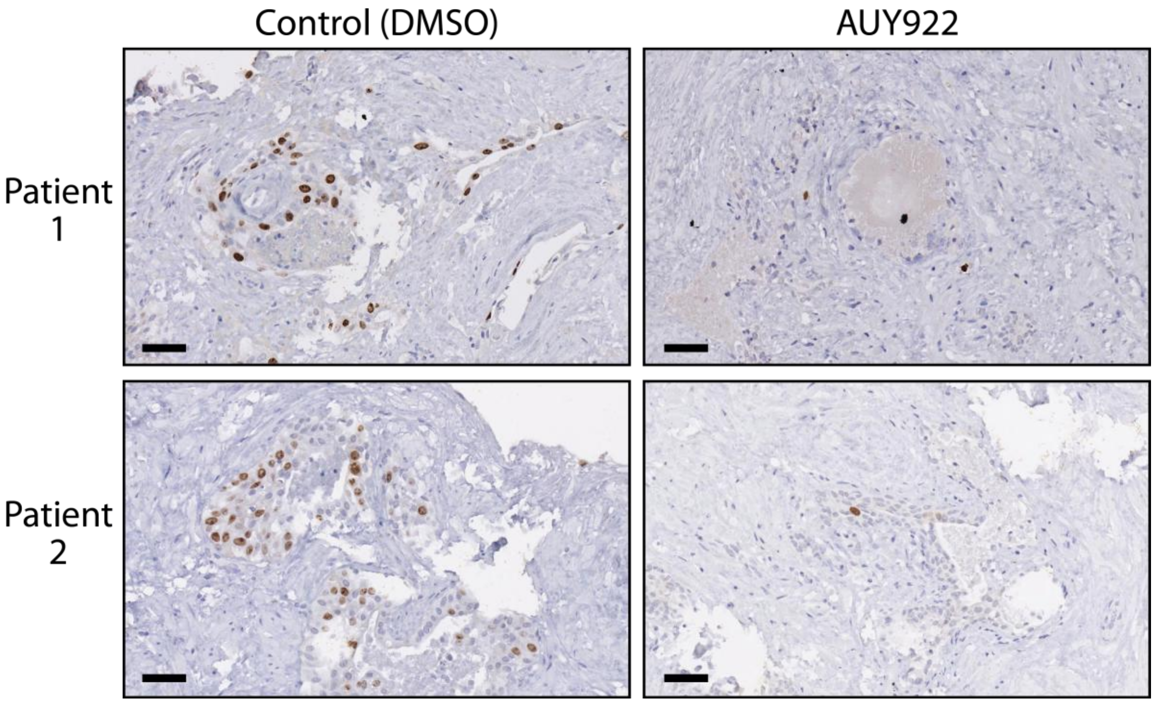

Supplementary Figure 5. Representative Ki67 immunostaining in *ex vivo* cultured prostate tumors treated with AUY922 at 20x magnification. Bar = 50 μm.
